# Exploring the Effects of Environmental Factors on the Development of 0–4-Year Old Children in The Netherlands

**DOI:** 10.3390/ijerph18157782

**Published:** 2021-07-22

**Authors:** Luuk van Wel, Paula van Dommelen, Moniek Zuurbier, Debbie Heinen, Jennie Odink, Janine Bezem, Paul H. Verkerk, Anjoeka Pronk, Gerard Hoek, Eelco Kuijpers

**Affiliations:** 1 Risk Analysis for Products in Development, The Netherlands Organisation for Applied Scientific Research TNO, P.O. Box 80015, NL-3508 TA Utrecht, The Netherlands; luuk.vanwel@tno.nl (L.v.W.); anjoeka.pronk@tno.nl (A.P.); 2 Department of Child Health, The Netherlands Organisation for Applied Scientific Research TNO, P.O. Box 3005, NL-2301 DA Leiden, The Netherlands; paula.vandommelen@tno.nl (P.v.D.); janine.bezem@vggm.nl (J.B.); paul.verkerk@tno.nl (P.H.V.); 3 Public Health Services Gelderland-Midden, P.O. Box 5364, NL-6802 EJ Arnhem, The Netherlands; moniek.zuurbier@vggm.nl (M.Z.); debbie.heinen@vggm.nl (D.H.); jennie.odink@vggm.nl (J.O.); 4 Institute for Risk Assessment Sciences (IRAS), Utrecht University, P.O. Box 80178, NL-3508 TD Utrecht, The Netherlands; g.hoek@uu.nl

**Keywords:** child health, early life development, environmental exposure, air pollution, green space, D-score

## Abstract

Air pollution, noise, and green space are important environmental exposures, having been linked to a variety of specific health outcomes. However, there are few studies addressing overall early life development. To assess their effects, associations between developmental milestones for a large population of 0–4-year old children in The Netherlands and environmental exposures were explored. Developmental milestones and background characteristics were provided by Preventive Child Health Care (PCHC) and supplemented with data from Statistics Netherlands. Milestones were summarized and standardized into an aggregate score measuring global development. Four age groups were selected. Environmental exposures were assigned to geocoded addresses using publicly available maps for PM_2.5_, PM_10_, PM_coarse_, NO_2_, EC, road traffic noise, and green space. Associations were investigated using single and multiple-exposure logistic regression models. 43,916 PCHC visits by 29,524 children were available. No consistent associations were found for air pollution and road traffic noise. Green space was positively associated in single and multiple-exposure models although it was not significant in all age groups (OR 1.01 (0.95; 1.08) (1 year) to 1.07 (1.01; 1.14) (2 years)). No consistent associations were found between air pollution, road traffic noise, and global child development. A positive association of green space was indicated.

## 1. Introduction

Our physical environment exposes us to numerous factors affecting our health on a daily basis. The World Health Organization (WHO) states that children are more susceptible for these factors, as they are constantly growing and developing [1]. The Global Burden of Disease Study has shown that significant health benefits can be achieved by reducing environmental exposures [2]. As burdens afflicted and benefits gained early on in life will have long lasting impacts, we would ideally decrease the impact of environmental exposures in very young children where positive changes will have the longest impact. Air pollution, road traffic noise, and green space are important environmental exposures in modern urban settings, having been linked to a variety of specific health outcomes [3]. Air pollution and road traffic noise are spatially associated, with increases in road traffic leading to increased air pollution and noise exposure [4]. More green space is correlated with lower air pollution and road traffic noise levels, especially due to less traffic and other sources in green areas [5]. It is therefore important to explore the effects of air pollution, road traffic noise and green space on health together. Few studies have addressed overall early life development however. A recent birth cohort study amongst Dutch school-age children suggested an association between air pollution exposure during fetal life and alterations in the cerebral cortex, mediating impaired inhibitory control, which could have long-term consequences [6]. Earlier studies have suggested negative relations between air pollutant exposure in children and impairment of brain development [7]. A systematic review performed for the WHO environmental noise guidelines assessed effects between environmental noise and cognition, all in child populations, finding little evidence but also recommending that more robust studies are required on this subject [8]. Contrary to air pollution and road traffic noise, green space may have beneficial effects on health, with a recent systematic review by Vanaken et al. finding beneficial associations in relation to emotional and behavioural effects in children, and also after adjustment for socio-economic and demographic confounders [9].

While there is some evidence linking environmental exposure to specific physical and mental health outcomes, few studies have assessed more comprehensive health outcomes such as overall development of children. Using a composite indicator in studying the effects of environmental exposures such as air pollution is particularly valuable, as these may be linked to a variety of both physical and mental health effects.

In The Netherlands, Preventive Child Health Care (PCHC) routinely monitors the development of almost all children, reaching >95% of 0–4-year old children [10]. During the first four years, there are thirteen scheduled visits. During these visits, PCHC professionals (physicians and nurses among others) evaluate child development. The Dutch Development Instrument (DDI, in Dutch: Van Wiechenonderzoek) is the standard instrument used to measure development from 0 to 4 years. The DDI consists of 75 milestones covering three domains of child development: fine motor activity, adaptive, personal/social behaviour, communication, and gross motor activity. The developmental score (D-score) is determined from an algorithm that summarizes the scores of the developmental milestones (pass/fail) into a single aggregate score measuring global development. It is a validated, unified indicator for early life development [11]. The advantage of using the D-score as a single measure to explore environmental exposure influence is that the D-score is on an interval scale, making it possible to rank the ability of individuals, groups or populations from low to high.

Whereas many (birth) cohort studies are limited to detailed information on relatively few children with developmental data, using regularly collected data from PCHC will enable the assessment of environmental exposures on the development of larger and less selected groups of children. This study will use PCHC data from one out of 25 regions in the Netherlands, with future possibilities of applying the methodology to all PCHC regions.

The aim of our study is to explore relations between long-term air pollution, road traffic noise, and green space exposure and global child development in a large population of 0–4-year old children.

## 2. Methods

### 2.1. Study Design and Population

For this study, data from PCHC region Gelderland Midden, Statistics Netherlands, and the Dutch National Georegister were used. The PCHC region Gelderland Midden is one of twenty-five regions, is located in the eastern part of The Netherlands and contains both urban and rural areas (Supplementary Materials Figure S1). PCHC provided data on all visits made by 0 to 4-year old children between October 2017 and October 2019, including scores of individual items on the DDI, age at time of assessment, sex, parental education, ethnicity, and the current residential address. Only children with a residential address within the region Gelderland-Midden were included: children visiting from an address outside of the study region were excluded. Earlier data were not readily available due to the introduction of a new digital registration system in October 2017. The data were supplemented by data from Statistics Netherlands on median standardized household incomes (2017) based on the neighborhood in which the residential addresses were located [12]. Geographic coordinates were extracted per address and per visit for estimating the environmental exposure at the time of each visit. DDI scores for each visit were converted into the D-score and subsequently standardized into the age-standardized D-scores (DAZ), which is explained in more detail below.

This study was approved by the Data Protection Officer of Children’s Healthcare in the PCHC region Gelderland Midden. Only researchers from Children’s Healthcare were able to access residential addresses, extracted geographical coordinates and other personally identifiable information. All data were linked and anonymized within PCHC, leaving an anonymous dataset with only DDI scores, characteristics, and environmental exposure estimates per visit to perform the main analysis upon.

### 2.2. Geocoding and Environmental Factors

Residential addresses were geocoded using the OpenStreetMap Nominatim API [13], which resulted in geographical coordinates for each address. The coordinates were then used to extract annual average levels for particulate matter (PM_2.5_, PM_10_, and PM_coarse_), nitrogen dioxide, elemental carbon, road traffic noise, and surrounding green from publicly available geospatial data for each residential address in the dataset. Most available maps contained yearly average exposure levels for 2017, with the exception of the surrounding green map (2016). As these were the most recent maps available at the time of analysis, exposure levels for addresses associated with visits in 2018 and 2019 were also based on these maps. As spatial contrasts appeared to be relatively stable in The Netherlands this was considered acceptable [14]. All geodata were processed using R (Version 3.6.2.) [15].

#### 2.2.1. Air Pollution

Yearly averaged exposure level maps of particulate matter (PM) fractions with a diameter of ‘2.5 microns or less’ (PM_2.5_) [16] and ‘10 microns or less’ (PM_10_) [17], nitrogen dioxide (NO_2_) [18], and elemental carbon (EC) [19] were downloaded from the Dutch National Georegister (NGR). In summary, the National Institute for Public Health and the Environment (RIVM) created these maps by combining their GCN national 1 × 1 km concentration maps (containing both measurement and dispersion model estimates) with dispersion modelling of local traffic information from the Dutch National Collaboration Programme on Air Quality (NSL) [20]. Estimates were made at the building level and subsequently averaged over grids of 25 × 25 m. The resulting publicly available 25 × 25 m maps for PM_2.5_ and PM_10_ were used here. The coarse fraction of particulate matter (PM_coarse_) was calculated per address by subtracting PM_2.5_ from PM_10_.

#### 2.2.2. Road Traffic Noise

The Dutch National Georegister contained noise level maps on various sources, including air, rail, and road traffic. For this study the road traffic noise map was used, containing yearly average day-evening-night levels (L_den_) in decibels [21]. Model estimates (using RMV2012 methodology [22]) by the RIVM were based on all national, provincial and municipal roads, resulting in a map with a resolution of 10 × 10 m.

#### 2.2.3. Surrounding Green

The surrounding green map was created by RIVM by combining national height maps (AHN2, AHN3), the building footprint map (BAG), and infrared imagery (CIR) taken during the summer [23]. The resulting map has a resolution of 10 × 10 m and shows the percentage of green (0–100%). Two values for surrounding greenspace were calculated for each residential address by drawing a circle (500 and 1000 m respectively) with the residential address at its centre point (i.e., a buffer). The average within the area covered by the circle was taken as the surrounding greenspace.

### 2.3. DAZ Score

The D-score is determined from an algorithm (Expected a posteriori (EAP) method) that summarizes the age-dependent developmental milestones (75 in total) into an age-dependent single aggregate score measuring global development [11]. In this study, the D-score is determined at the visits at ages one, two and, three years and three years and nine months.

Since the D-score increases with age, it is important to adjust this score for an age-dependent reference (similar to the growth charts). Age-conditional references of the D-score were previously developed by the LMS method [11]. With these references, DAZ scores were calculated. The DAZ in the general population has a mean of zero and a standard deviation of one. This implies that if a child has, for example, a DAZ of −1, his or her development is 1 SD below the median of the population; 84% of the population has a higher developmental score and 16% has a lower developmental score than this child. With the current use of the age-specific milestones, approximately 90% of the children pass for each milestone. In daily practice, milestones for the next visit are usually not evaluated in children who have a more advanced development. As a consequence, the DAZ shows a ceiling effect. We, therefore, categorized the DAZ into 2 levels: <−1 and ≥−1. A cut-off point of −1 was chosen in order to define a subgroup with a relatively low global developmental score.

### 2.4. Statistical Analysis

To investigate whether the associations between environment and development change over time, we selected the following four regular visits to PCHC: one year, two years, three years, and three years and nine months. A range of ±3 months surrounding these ages was taken to account for slightly earlier or later appointments. In the rare case that a child would have more than one visit logged in the three-month period, only the visit closest to the desired age (e.g., one year (twelve months)) was kept, dropping all other visits in the three month range for said child.

Correlations between environmental factors were explored using Spearman’s Rank correlation coefficient. Logistic regression analyses were performed to explore relations between environmental factors and global child development separately for the visits at ages one, two, three years, and three years and nine months, using the categorized DAZ (<−1 vs. ≥−1) as the dependent variable. We modelled the occurrence of a positive DAZ (≥−1), indicating optimal global development. Independent variables were the environmental factors air pollutants, road traffic noise, and surrounding green standardized to z-scores, so that the effect sizes between the factors can be compared. Two single-exposure models were defined, including the environmental factors univariate, with model 1 adjusting for age and sex of the child and model two adjusting for all potential confounders (age of the child, sex, median neighborhood household income, ethnicity, and parental education). One multiple-exposure model was defined (Model 3), including the environmental factors multivariate and adjusting for all aforementioned potential confounders. Parental education was categorized into low (elementary or secondary), medium (vocational) or high (college, university) for the highest educated parent. Ethnicity was divided into Dutch, western or non-western, which was based on the country of origin of the parents and the child, following Statistics Netherlands criteria [24]. These criteria define a person with a western migration background as originating from a country in Europe (excluding Turkey), North America and Oceania, or from Indonesia or Japan. The latter are considered western by Statistics Netherlands due to their socioeconomic and cultural position. These variables were available on the individual level (i.e., for each child). Standardized household incomes (2017) from Statistics Netherlands were available on the neighborhood level (using four-digit postal codes) using the categories “<40th percentile, 40th ≤ 60th percentile, or ≥60th percentile of households in the Netherlands. SPSS version 25 was used for these analyses [25].

## 3. Results

Information on DDI, age at time of visit, sex, median neighborhood household income, and all environmental factors was available for 51,736 visits to PCHC, which were made by 34,913 children. Excluding children with missing values on parental education or ethnicity, 43,916 visits made by 29,524 children were available. Most visits fell in the one year age group (12.918), followed by 9919, 9849, and 11,230 visits respectively for 2 years, 3 years, and 3 years 9 months. More than half (16,263 children) contributed only one visit to the dataset, with 12.130 and 1131 children contributing two and three visits respectively (a flowchart is provided in Supplementary Materials Figure S2). 51% of all included children were boys. The majority of children (55.6%) had at least one parent which received a high education, 82.9% of children possessed the Dutch nationality and the majority of children (72.1%) lived in a neighborhood with households ranking in the 40th–60th percentile income bracket. Full characteristics of all included children are given in Table 1.

### 3.1. Environmental Factors and DAZ

Mean levels over all visits were 11.4 µg/m^3^ PM_2.5_, 18.8 µg/m^3^ PM_10_, 7.4 µg/m^3^ PM_coarse_, 19.5 µg/m^3^ NO_2_, 0.8 µg/m^3^ EC, 52.2% green space in 500 m buffer, 59.0% green space in 1000 m buffer, and 50.8 dB road traffic noise. Exposure levels were nearly identical for boys and for girls, but varied when stratified for parental education, ethnicity, and median household income, with lower socio-economic factors having higher air pollutant and road noise levels and less surrounding green space (Table 2). Variation in environmental factors is largest between the median household income categories, despite this being a factor at the neighborhood level rather than the individual level. The variation in the environmental factors was lowest for the particulate matter fractions (PM_2.5_, PM_10_, PM_coarse_) and highest for surrounding green space both overall and in all stratified categories. The interquartile ranges (25th–75th percentile) follow a similar pattern, varying only a few units surrounding the mean. Looking at the distribution, the spread was large with a right-skewed distribution. Visual representations are given as box plots in Supplementary Materials Figure S3.

The majority (86.4%) of all children obtained a DAZ ≥ −1 (indicating a relatively normal to high global developmental score). This proportion varied greatly stratified for sex (boys, 83.3%), parental education (low education, 80.0%), ethnicity (non-western, 82.1%), and household income (<40th percentile, 83.9%) (Table 2). Stratified for age, the percentage of children with a DAZ ≥ −1 was 88.9%, 79.8%, 86.2% and 87.8% for 1 year, 2 years, 3 years, and 3 years 9 months, respectively. Sub groups of children with lower parental education, non-Dutch ethnicity, and lower household income had a larger percentage of a relatively low global developmental score. Simultaneously, these potential confounding factors were related to the environmental exposures in the expected pattern, e.g., air pollution being generally lower and the amount of surrounding green space being higher for the high education, high income, Dutch nationality groups, indicating significant potential for confounding.

### 3.2. Correlations and Selection of Environmental Factors

**Figure 1:**
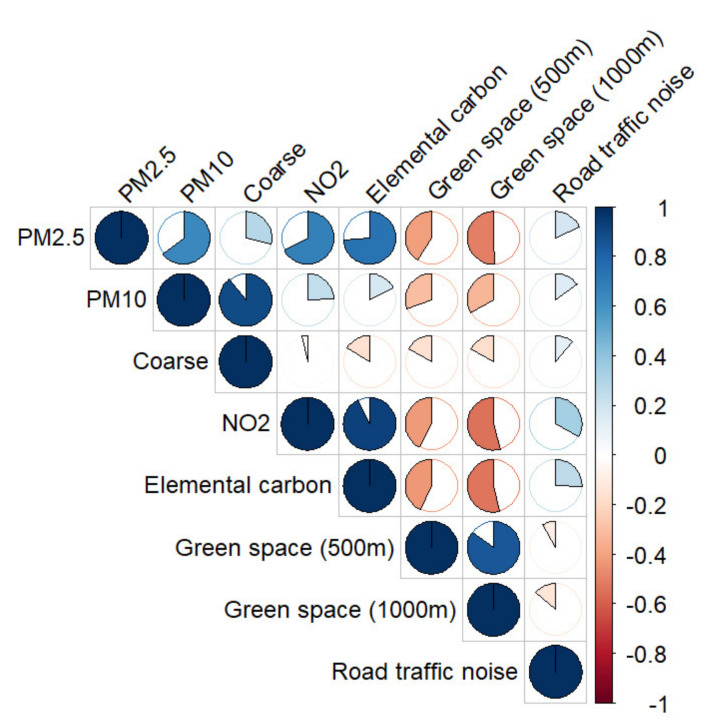
Spearman correlations between PM_2.5_, PM_10_, PM_coarse_, NO2, elemental carbon, green space (500 m and 1000 m buffer), and noise. Clockwise (blue) denotes positive correlations, counter clockwise (red) denotes negative correlations. N = 43.916 visits.

Environmental factors at the residential addresses of the children were mostly moderately correlated, with air pollution being positively correlated with road traffic noise (L_den_) and negatively correlated with green space (Figure 1). Highest correlations were seen between PM_10_ and PM_coarse_ (0.89), PM_2.5_ and elemental carbon (EC) (0.74), PM_2.5_ and NO_2_ (0.68), PM_10_ and PM_2.5_ (0.66), PM_2.5_ and green space (1000 m) (−0.50), and both NO_2_ and EC and green space (1000 m) (each −0.53). Following these results, multiple environmental factors were selected for further analyses. For air pollution, NO_2_, PM_2.5_, and PM_coarse_ were included. The correlation between the latter was limited (0.31) and together these factors cover a wide spectrum of PM fractions, whereas PM_10_ by definition includes the PM_2.5_ fraction as well. Elemental carbon was excluded due to its overlap and subsequent high correlation with NO_2_ as an indicator of road traffic and its high correlation with PM_2.5_. Green space within 500 m was highly correlated with green space within 1000 m and (slightly) less correlated to other factors compared to green space within 1000 m in the collected data, therefore we chose to exclude green space within 1000 m from the analyses. Lastly, road traffic noise was included as a factor representing noise pollution originating from traffic.

**Table 2:** Environmental factors and DAZ stratified per sex, ethnicity, parental education, and household income. Values are arithmetic means (standard deviation) unless stated otherwise. N represents the number of children included.

Characteristic	N	DAZ ^A^ ≥ −1	PM_2.5_	PM_10_ ^B^	PM_coarse_	NO_2_	EC ^B^	Green Space	Green Space ^B^	Road Traffic Noise
	Children	%	µg/m^3^	µg/m^3^	µg/m^3^	µg/m^3^	µg/m^3^	% (500 m)	% (1000 m)	dB
Overall	29,524	86.4	11.4 (0.4)	18.8 (0.9)	7.4 (0.8)	19.5 (2.5)	0.8 (0.1)	52.2 (12.2)	59.0 (11.1)	50.9 (5.5)
Sex										
Boys	15,061	83.3	11.4 (0.4)	18.8 (0.9)	7.4 (0.8)	19.5 (2.5)	0.8 (0.1)	52.2 (12.2)	59.1 (11.1)	50.8 (5.5)
Girls	14,463	89.7	11.4 (0.4)	18.8 (0.9)	7.4 (0.8)	19.5 (2.5)	0.8 (0.1)	52.1 (12.2)	59.0 (11.1)	50.9 (5.4)
Parental education										
Low	2544	80.0	11.5 (0.4)	19 (1.0)	7.6 (0.9)	19.7 (3.1)	0.8 (0.1)	50.8 (13.3)	57.9 (12.3)	51.1 (5.6)
Medium	10,557	85.2	11.4 (0.4)	18.9 (1.0)	7.5 (0.9)	19.3 (2.6)	0.8 (0.1)	52.0 (12.6)	59.1 (11.6)	50.7 (5.4)
High	16,423	88.2	11.4 (0.4)	18.7 (0.9)	7.3 (0.7)	19.7 (2.4)	0.8 (0.1)	52.5 (11.7)	59.2 (10.5)	50.9 (5.5)
Ethnicity										
Dutch	24,467	87.1	11.4 (0.4)	18.8 (1.0)	7.4 (0.8)	19.3 (2.4)	0.8 (0.1)	52.9 (12.3)	59.8 (11.0)	50.7 (5.5)
Non-western	3453	82.1	11.6 (0.4)	18.8 (0.7)	7.3 (0.5)	20.9 (2.9)	0.8 (0.1)	47.7 (10.4)	54.3 (10.3)	51.8 (5.4)
Western	1604	85.2	11.5 (0.4)	18.7 (0.8)	7.2 (0.5)	20.2 (2.6)	0.8 (0.1)	50.5 (11.9)	57.1 (10.7)	51.3 (5.6)
Household income ^C^										
<40th	3550	83.91	11.6 (0.4)	18.7 (0.5)	7.1 (0.2)	22.5 (3.0)	0.9 (0.1)	47.5 (11.4)	53.2 (10.4)	52.3 (5.5)
40th–60th	21,289	86.5	11.4 (0.4)	18.8 (1.0)	7.5 (0.8)	19.0 (2.2)	0.8 (0.1)	52.2 (12.2)	59.2 (11.3)	50.6 (5.4)
>60th	4685	88.0	11.4 (0.4)	18.7 (1.0)	7.3 (0.7)	19.6 (2.1)	0.8 (0.1)	55.5 (11.5)	62.6 (8.6)	50.9 (5.4)

^A^ Standardized (by age) score measuring global child development. ^B^ PM_10_, elemental carbon (EC) and green space (1000 m buffer) were dropped from further analyses based on their correlations with other environmental factors. ^C^ Median standardized neighborhood household income, percentile in relation to all households in The Netherlands.

### 3.3. Single-Exposure Logistic Regression Models

We hypothesized a negative association (odds ratio (OR) < 1) with a medium/high developmental score (DAZ ≥ −1) for PM2.5, PM_coarse_, NO_2_, and road traffic noise, and a positive association (OR > 1) for green space. Model 1 shows relatively large associations of environmental factors on DAZ. The direction of these associations varies strongly between age groups however, with little consistency to be found. Overall Model 1, the least complex model, shows little consistency across age groups and with the hypothesis (Table 3). When including the other suspected confounding factors (Model 2), attenuated towards unity, suggesting important confounding. Nearly all negative associations disappear. PM2.5, NO_2_ and road traffic noise have mostly consistent positive associations over age groups, opposite to the hypothesized direction. Odds ratios were small and generally highly non-significant. PM_coarse_ has (hypothesized) a negative association with development for age groups one and two years, and a positive association at three years and three years and nine months (Table 3). Green space is positively associated with development for all age groups, though only borderline significantly so for age group two years.

### 3.4. Multiple-Exposure Logistic Regression Models

To adjust for effects of other correlated environmental factors, a multiple-exposure model including all other environmental factors was created (Model 3 in Table 3). This resulted in similar inconsistent associations over age groups for most environmental factors, with only small changes compared to the single-exposure model. Notable are PM_coarse_, which kept a consistent trend over all three models: negatively associated with development for age groups one and two years, positively associated for age groups three years and three years and nine months. It should be noted that only in age groups one and three years, nine months there was a significant association. These are also the age groups with the most visits included (12,918 and 11,230 respectively, versus <10,000 in other groups). Green space indicates a consistent positive association with development over all age groups, although only reaching significance for the oldest group (3 years, 9 months). Road traffic noise found negligible associations either way (Table 3).

**Table 3:** Association between global developmental score and environmental factors, stratified per age group. Values are Odds Ratios (95% confidence interval), expressing the odds of a positive developmental score (medium/high, DAZ ≥ −1) for a one unit change in the z-score of exposures. N represents the number of children (visits) included in the model.

Environmental Factors	Single-Exposure Model 1 ^A^	Single-Exposure Model 2 ^B^	Multiple-Exposure Model 3 ^C^
Z-score PM_2.5_			
1 year (N = 12,918)	**1.07 (1.02;1.13)**	1.03 (0.98;1.09)	1.08 (0.99;1.17)
2 years (N = 9919)	**0.94 (0.89;0.99)**	1.01 (0.96;1.06)	1.03 (0.95;1.11)
3 years (N = 9849)	0.95 (0.89;1.01)	1.01 (0.95;1.08)	0.95 (0.86;1.05)
3 years, 9 months (N = 11,230)	0.96 (0.91;1.02)	1.04 (0.98;1.10)	1.00 (0.91;1.10)
Z-score PM_coarse_			
1 year (N = 12,918)	**0.89 (0.85;0.94)**	**0.92 (0.88;0.97)**	**0.90 (0.85;0.96)**
2 years (N = 9919)	0.97 (0.93;1.02)	0.97 (0.92;1.02)	0.96 (0.90;1.02)
3 years (N = 9849)	1.04 (0.98;1.10)	1.05 (0.99;1.12)	1.07 (1.00;1.15)
3 years, 9 months (N = 11,230)	**1.09 (1.02;1.16)**	**1.09 (1.02;1.16)**	**1.09 (1.01;1.17)**
Z-score NO_2_			
1 year (N = 12,918)	**1.13 (1.07;1.20)**	1.06 (1.00;1.13)	1.00 (0.90;1.10)
2 years (N = 9919)	**0.93 (0.88;0.98)**	1.02 (0.97;1.08)	1.03 (0.94;1.12)
3 years (N = 9849)	0.95 (0.90;1.01)	1.03 (0.97;1.10)	1.10 (0.99;1.21)
3 years, 9 months (N = 11,230)	**0.93 (0.88;0.99)**	1.03 (0.97;1.10)	1.04 (0.94;1.15)
Z-score green space (500 m)			
1 year (N = 12,918)	0.95 (0.90;1.00)	0.98 (0.93;1.03)	1.01 (0.95;1.08)
2 years (N = 9919)	**1.11 (1.06;1.17)**	1.05 (0.99;1.10)	**1.07 (1.01;1.14)**
3 years (N = 9849)	**1.08 (1.01;1.14)**	1.02 (0.96;1.08)	1.03 (0.96;1.10)
3 years, 9 months (N = 11,230)	**1.09 (1.03;1.16)**	1.02 (0.96;1.09)	1.04 (0.97;1.11)
*Z-score road traffic noise*			
1 year (N = 12,918)	1.04 (0.98;1.09)	1.02 (0.96;1.07)	1.01 (0.96;1.08)
2 years (N = 9919)	0.99 (0.94;1.04)	1.01 (0.97;1.07)	1.01 (0.96;1.07)
3 years (N = 9849)	0.98 (0.93;1.04)	1.00 (0.95;1.07)	0.99 (0.92;1.05)
3 years, 9 months (N = 11,230)	1.02 (0.96;1.08)	1.05 (0.99;1.11)	1.03 (0.97;1.10)

^A^ Outcome: dichotomized DAZ, environmental factor as independent variable, corrected for age and sex. ^B^ Outcome: dichotomized DAZ, environmental factor as independent variable, corrected for age, sex, ethnicity, parental education, and neighborhood median household income. ^C^ Outcome: dichotomized DAZ, environmental factor as independent variable, corrected for age, sex, ethnicity, parental education, neighborhood median household income, and all other environmental factors.

## 4. Discussion

### 4.1. Main Findings

We have found no consistent associations between global development in 0–4-year old children (DAZ) and environmental factors. A positive association of surrounding green space with developmental score in all age groups was indicated, but only reached statistical significance in one age group (two years).

### 4.2. Comparison with Previous Studies

To our knowledge no other studies have specifically assessed associations with global development in 0–4-year old children. A recent review looking at particulate exposure during pregnancy found a small reduction in birthweight, while noting that most included studies were unable to correct for socio-economic factors and maternal smoking [26]. A different literature review found associations between air pollution and cognitive impairment in studying children and adolescents older than four years, often involving academic performance tests as cognitive measures [7]. The quality of exposure and outcome measures in the reviewed studies were noted as key limitations, which our study addressed by combining residential address level models with the DAZ-score derived from systematically collected data on child development. Still, we found no consistent associations between early life development and air pollution, despite these effects being suggested in studies in older children. Possible explanations entail insufficient correction for potential confounders (e.g., socio-economic factors, prenatal exposures) which might lead to spurious associations.

Noise exposure was often assessed at schools for school age children and adolescents, such as in the cross-national RANCH study [27]. Indications that aircraft noise could negatively affect cognitive development were found there, but not for road traffic noise. In our study we explored road traffic noise at the residential address, but similarly could not find an association on development in any age category. It is possible that road traffic noise does not impair development in these early years or that the effect is mediated by socio-economic factors, although our models correcting for ethnicity, parental education, and household income did not provide an indication for this.

Although no studies focusing on green space and 0–4-year old children were found, studies on mental health in older children and adolescents indicate that it would be of interest to assess different green space exposure concepts and ranges, with a Spanish study finding a beneficial effect of outdoor surrounding but not residential greenness on cognitive development of school children [9,28]. A study on the effect of green space definition on associations with overweight and physical activity similarly noted the importance of definition [29]. Our study did not distinguish between various types of green space and types of associated uses (e.g., social activities, physical exercise) and the question remains which mechanisms might positively affect global development in the 0–4-year old age group. Different methodologies for assessing relevant surrounding green might be of interest in further exploration of the effects of green space, for example our current methodology included private gardens but excluded paved urban play spaces.

### 4.3. Strengths and Weaknesses

A strength of our study is that information on a large set of developmental milestones was obtained from nearly 44,000 standardized health visits of nearly 30,000 0–4-year old children. The collection of this information by PCHC is well organized in the Netherlands, including almost all (>95%) children in the study region and enabling us to investigate environmental factors with potential small effects. Combining the milestones into the composite D-score gave us the opportunity to study quantitative effects of environmental factors on early childhood development across several age groups.

Another strength is that, thanks to publicly available high-resolution environmental exposure maps, we were able to assess modelled environmental factors at the residential address level, allowing for high resolution spatial variations in exposure to be included. While a single map per environmental exposure (in 2016 or 2017) was used for all age categories, rather than one map per year that the visit took place, we do not believe this will be a major influence as variation in annual average environmental factors at the address level between a few years is expected to be mostly limited. It should be noted that local sources (e.g., noise from air-conditioning units, indoor particulate matter sources) are not taken into account by these maps. Neither is the amount of time spent at home versus at school or other locations. Also, a strength of our study is that we were able to include high quality information on socio-economic factors in our models to adjust the effects for these influences, while many other studies were not able to adjust for these potential confounders. However, it is unclear whether other potentially important background characteristics (at child-level) (e.g., smoking, preschool attendance) would have influenced the effects that we found in our study.

A limitation of the PCHC visits is the fact that parents do not always attend all scheduled visits (i.e., these are not mandatory) and that PCHC professionals do not always register all milestones during a visit (i.e., developmental milestones ahead of what the child should demonstrate at a certain age are not marked in advance) [30]. It should be noted that we had to transform the DAZ into two categories (low versus high), since by definition approximately 90% of the children pass the milestones at the visits, which creates a ceiling effect. This may have reduced the information that is available for development and, consequently, may have decreased our ability to detect the actual effect of environmental factors on development. Moreover, the D-score was developed as a screening instrument, facilitating interpretation of children’s abilities across different ages (just as centim are used for height), and enabling comparisons of children’s development both within and between countries [11]. Up until now, the D-score has never been used as an outcome in environmental effects studies. It may be that the D-score is not able to detect small effects, because of some limitations. The total score depends not only on the actual developmental status of the child, but also on the set of milestones administered, domains are difficult to separate, the ceiling effect, and the lack of knowledge on the inter and intra-rater reliability of the DDI. On the other hand, the D-score did show expected associations with background characteristics (e.g., higher parental educational level resulting in less children with a relatively low score). These results suggest that it may be feasible to detect significant associations between the D-score and outcomes.

## 5. Conclusions

Using a large population with high quality data on child development and environmental exposures, our study found no consistent associations between air pollution, road traffic noise, and the global development of 0–4 year-old children. A positive association of green space was indicated.

## Figures and Tables

**Table 1:** Characteristics of included children.

Characteristic	N	Percentage (%)
N	29,524	100
Sex		
Boys	15,061	51.0
Girls	14,463	49.0
Parental education		
Low	2544	8.6
Medium	10,557	35.8
High	16,423	55.6
Ethnicity		
Dutch	24,467	82.9
Non-western	3453	11.7
Western	1604	5.4
Household income ^A^		
<40th	3550	12.0
40th–60th	21,289	72.1
>60th	4685	15.9

^A^ Median standardised neighbourhood household income, percentile in relation to all households in the Netherlands.

## Data Availability

Maps of environmental factors are openly available in the National Georegister (http://nationaalgeoregister.nl/, accessed on 23 October 2020). Other data is available from the corresponding author on reasonable request.

## Supplementary Materials

The following are available online at https://www.mdpi.com/article/10.3390/ijerph18157782/s1, Figure S1: Study area, Figure S2: Flowchart, Figure S3: Boxplots of all environmental factors
