# Supplementary material for: Exploring the Effects of Environmental Factors on the Development of 0–4-Year Old Children in The Netherlands
Source: Int J Environ Res Public Health. 2021 Jul 22;18(15):7782. doi: 10.3390/ijerph18157782 (PMC8345559; doi:10.3390/ijerph18157782)
Supplement: Supplementary file 1 [file ijerph-18-07782-s001.zip › ijerph-1293263 Supplementary Materials.pdf]

SUPPLEMENTARY MATERIALS

Figures

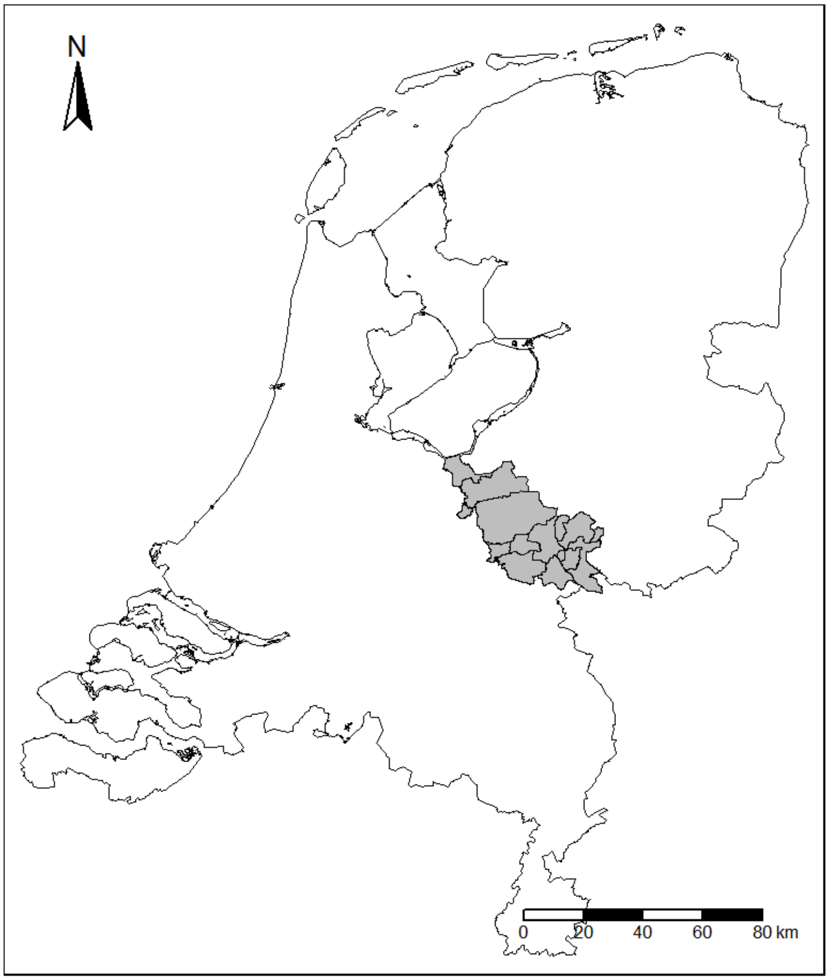

Figure S1. Study area in grey

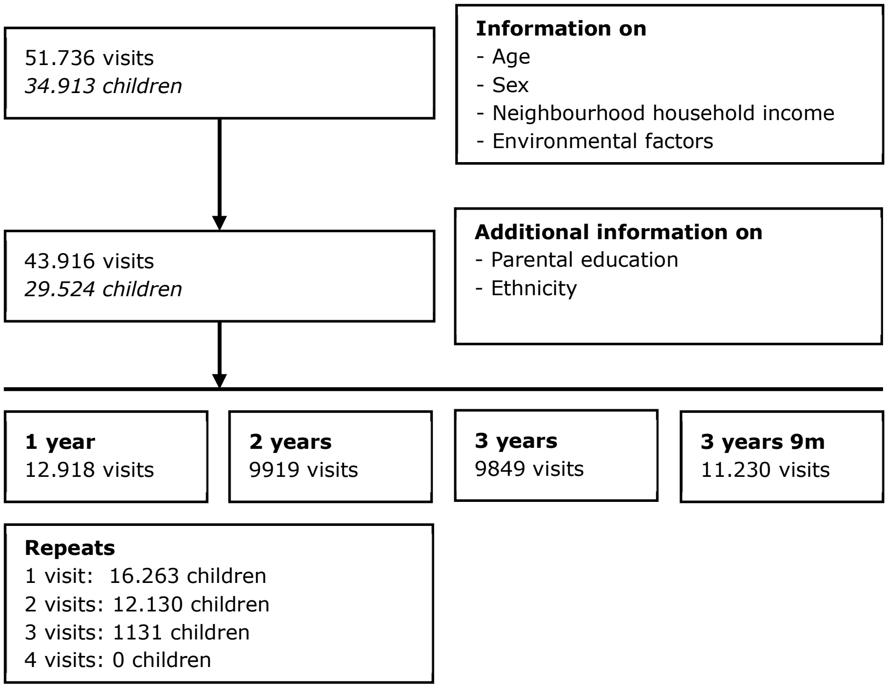

Figure S2. Flowchart. There were no children with visits in all four age categories, as this was not possible within the time period included in this study (October 2017 – October 2019).

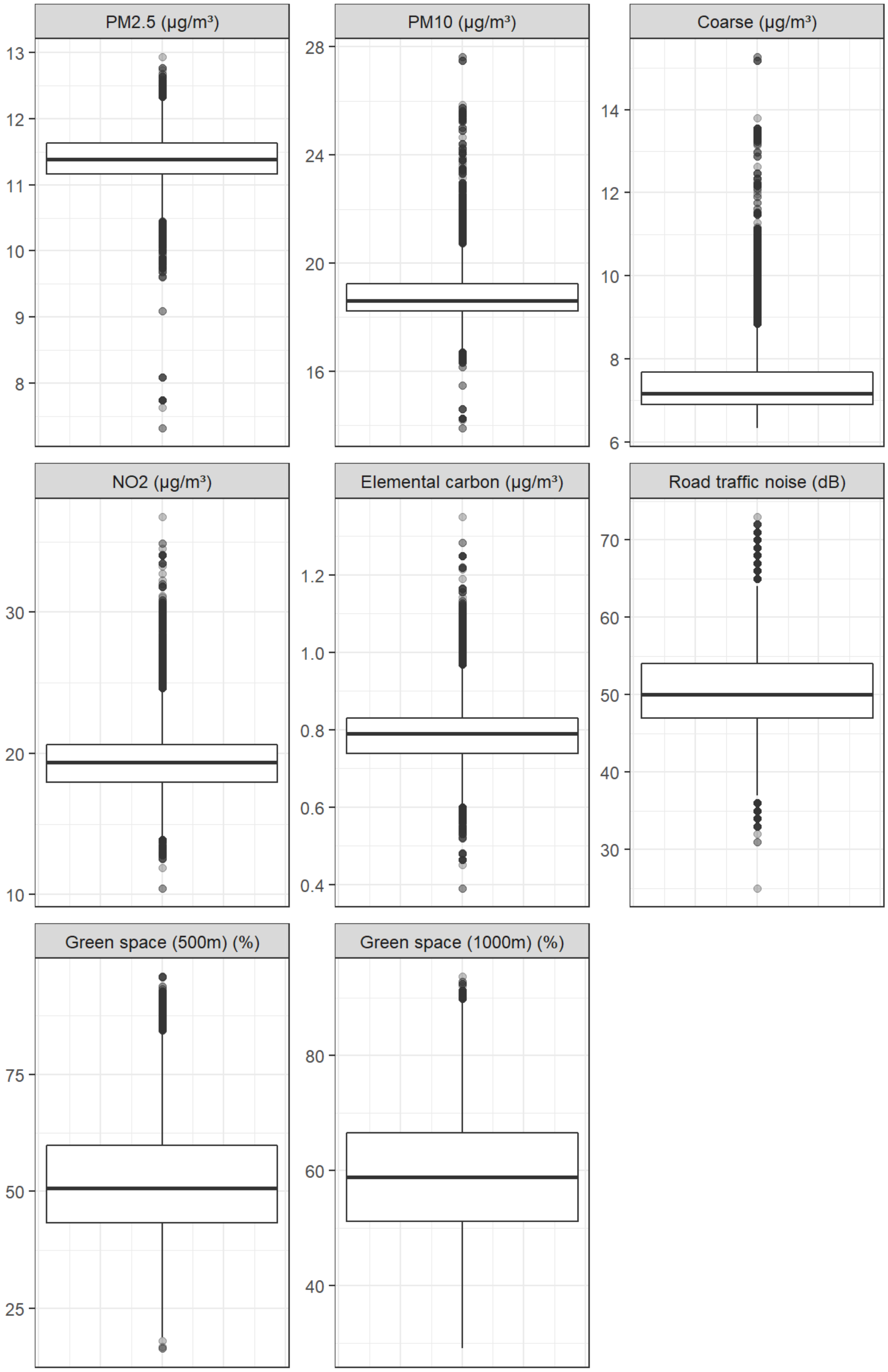

Figure S3. Boxplots of all environmental factors representing annual average air pollution and road traffic noise of 2017. Green space represents summer averages for 2017. The length of the box represents the 25th to 75th percentile (interquartile range) with the horizontal line within the box representing the median.Outliers are represented by dots, with the colour of the dots representing the amount of dots in that place (darker equals more dots).
